# An Information System for European culture collections: the way forward

**DOI:** 10.1186/s40064-016-2450-8

**Published:** 2016-06-17

**Authors:** Serge Casaregola, Alexander Vasilenko, Paolo Romano, Vincent Robert, Svetlana Ozerskaya, Anna Kopf, Frank O. Glöckner, David Smith

**Affiliations:** Micalis Institute, INRA, AgroParisTech, CIRM-Levures, Université Paris-Saclay, 78350 Jouy-en-Josas, France; All-Russian Collection of Microorganisms, IBPM RAS, Pushchino, Russia; IRCCS AOU San Martino IST, Largo R. Benzi 10, 16132 Genoa, Italy; The Centraalbureau voor Schimmelcultures (CBS) Fungal Biodiversity Centre, Utrecht, The Netherlands; Jacobs University Bremen, Bremen, Germany; Max Planck Institut for Marine Microbiology, Bremen, Germany; CABI, Bioservices, Egham, Surrey TW20 9TY UK

**Keywords:** Culture collection, Microorganism, Information Technology, Microbial domain Biological Resource Centre

## Abstract

Culture collections contain indispensable information about the microorganisms preserved in their repositories, such as taxonomical descriptions, origins, physiological and biochemical characteristics, bibliographic references, etc. However, information currently accessible in databases rarely adheres to common standard protocols. The resultant heterogeneity between culture collections, in terms of both content and format, notably hampers microorganism-based research and development (R&D). The optimized exploitation of these resources thus requires standardized, and simplified, access to the associated information. To this end, and in the interest of supporting R&D in the fields of agriculture, health and biotechnology, a pan-European distributed research infrastructure, MIRRI, including over 40 public culture collections and research institutes from 19 European countries, was established. A prime objective of MIRRI is to unite and provide universal access to the fragmented, and untapped, resources, information and expertise available in European public collections of microorganisms; a key component of which is to develop a dynamic Information System. For the first time, both culture collection curators as well as their users have been consulted and their feedback, concerning the needs and requirements for collection databases and data accessibility, utilised. Users primarily noted that databases were not interoperable, thus rendering a global search of multiple databases impossible. Unreliable or out-of-date and, in particular, non-homogenous, taxonomic information was also considered to be a major obstacle to searching microbial data efficiently. Moreover, complex searches are rarely possible in online databases thus limiting the extent of search queries. Curators also consider that overall harmonization—including Standard Operating Procedures, data structure, and software tools—is necessary to facilitate their work and to make high-quality data easily accessible to their users. Clearly, the needs of culture collection curators coincide with those of users on the crucial point of database interoperability. In this regard, and in order to design an appropriate Information System, important aspects on which the culture collection community should focus include: the interoperability of data sets with the ontologies to be used; setting best practice in data management, and the definition of an appropriate data standard.

## Background

 Information management is key to the operation and use of culture collections (CCs, see Table 1 for list of abbreviations); here we use CC as a generic term to include both the classical culture collection and the more recently defined microbial domain Biological Resource Centres (mBRCs). Information Systems for microbial genetic resources consist *a minima* of a website describing the CC, and a searchable electronic catalogue containing information on the microbial strains preserved by the CC. In addition, an appropriate Information System may also contain informatics tools to support the collections’ staff in curation activity and to provide users with information in a structured way. Information Systems also facilitate communication between users and CCs which plays an important role in the optimal exploitation of information and sourcing the right materials for research.

**Table 1:** List of acronyms

BRC: Biological Resource Centre
CC: Culture Collection
CABRI: Common Access to Biological Resources and Information
ECCO: European Culture Collections’ Organization
ELIXIR: European Life Science Infrastructure for Biological Information
EMbaRC: European Consortium of Microbial Resource Centres
EnvO: Environmental Ontology
ESFRI: European Strategy Forum on Research Infrastructures
GBIF: Global Biodiversity Information Facility
GBRCN: Global Biological Resource Centre Network
GCM: Global Catalogue of Microorganisms
IT: Information Technology
MEO: Metagenome and Microbes Environmental Ontology
MINE: Microbial Information Network Europe
MIRRI: Microbial Resource Research Infrastructure
NCBO: National Center for Biomedical Ontology
OBO: Open Biological and Biomedical Ontologies Foundry
OECD: Organization for Economic Co-operation and Development
R&D: Research and Development
SOP: Standard Operating Procedures
TDWG: Taxonomic Databases Working Group
WDCM: World Data Centre for Microorganisms
WFCC: World Federation of Culture Collections

For a number of years, electronic Information Systems were constructed according to various parameters, including the type and characteristics of the microorganisms, the needs and scientific interests of the curators, the availability of Information Technology (IT) tools, and the presence of IT staff. To improve the value and utility of the data offered, a European initiative, the Microbial Information Network Europe (MINE) was initiated in 1985 by CCs from Germany, the Netherlands, United Kingdom, Belgium and Portugal; several other CCs from other European countries subsequently joined MINE (Gams et al. 1990). MINE led to the construction of the first major multinational model for a common catalogue that covered all types of microorganisms, including viruses. This project facilitated a number of different CCs in Europe, including the Centraalbureau Schimmelcultures (CBS, The Netherlands), Deutsche Sammlung von Mikroorganismen und Zellkulturen Gmbh (DSMZ, Germany), CAB International (CABI, United Kingdom), Collection de Levures d’Intérêt Biotechnologique (CLIB, France), as well as others from 10 European countries to produce common interoperable catalogues. A key feature in this Information System was the definition of common minimal datasets comprising approximately 60 characteristics, including, e.g., taxonomy, physiological and biochemical properties, geographical origin, substrate of isolation, isolator, depositor (Gams et al. 1988; Stalpers et al. 1990).

Unfortunately, the short-term nature of the funding prohibited this initiative from keeping pace with the development of informatics tools and the initiative was thus progressively abandoned. Nevertheless, MINE had a very positive impact on European CCs; indeed, a number of them retain all, or part, of the MINE dataset in their catalogues to this day. MINE was a landmark in CC collaboration on database projects, but CCs were unable to capitalize on this initiative to truly harmonize their data handling and use. CCs have generally made their individual catalogues available on line making cross collection searches difficult. Only now are they re-engaging with each other in Europe and moving towards the creation of interoperable databases. This endeavour is greatly facilitated by the creation of the Microbial Resource Research Infrastructure (MIRRI, http://www.mirri.org/), a research infrastructure that is being designed in the context of the European Strategy Forum on Research Infrastructures (ESFRI).

Building on experiences such as MINE and the subsequent EU-funded project Common Access to Biological Resources and Information (CABRI, http://www.cabri.org/) (Romano et al. 2005), and enriched by the present survey of CC users and curators, this report describes how MIRRI can move towards an optimized Information System to facilitate access to the data from CCs at various levels. A number of the initiatives described here were conducted to better understand the users’ needs in order to prepare the structure of the MIRRI Information System. These initiatives comprise a user survey on the content and the extent of data retrieval from CCs, the monitoring of user requests on collection sites and integration systems, i.e., sites where information on the content from a number of CCs are integrated, and the definition of the required features of the portal for user access, both internal and external to the project.

## Consultation of CC users and CC curators

To date, the opinion of CC users has rarely been taken into consideration when designing CC Information Systems; CCs have relied upon the fact that managers and curators are also users of CCs and thus their feedback was considered to be sufficient. To remedy this shortfall, MIRRI polled the users of European CCs to determine their precise needs and thus identify the required content and capacity of the MIRRI Information System. The main European resource providers consulted can be divided into two groups: (1) public collections belonging to the European Culture Collections’ Organization (ECCO, http://www.eccosite.org/) and (2) research collections that conserve microbial strains that are currently not accessible to the wider community.

Resource providers and users were consulted through four questionnaire-based surveys:

1. The “ECCO-CC” questionnaire: survey of the public mBRCs/CCs that are members of ECCO
2. The “non-ECCO-CC” questionnaire: survey of the non-public CCs within laboratories of European research institutes, public health centres, universities, national reference laboratories and hospitals
3. The “User” questionnaire: survey of current and, potential, future users of microbial resources and related services
4. The “Innovative Services” questionnaire: survey of current and, potential, future users of microbial resources and services on the innovative aspects of MIRRI

The collection-targeted surveys were carried out in Spring 2013 among 60 of the ECCO member collections which generated an 80 % response rate. The user-targeted surveys were completed in March 2014 receiving nearly 1200 responses. The details of these surveys are available in a public document submitted to the European Commission as deliverable D2.1 “Compilation report on outputs from WP2 tasks 1–5, intermediate findings, conclusions and recommendations” (http://dx.doi.org/10.5281/zenodo.49678). Many of the responses were provided confidentially, and in such cases the source data are not included in the public report.

User feedback about needs and expectations related to the use of microbial resources and associated services was compiled, analysed, and evaluated, and will serve as the basis for the design and resultant output of MIRRI’s Information System. Two users groups, academic and industrial, were identified as having broadly different needs and expectations. Nevertheless, together, they were in agreement about the need to broaden public CC holdings and services to improve access to microbial expertise and data, and for the need to introduce mechanisms to optimize the MIRRI’s genetic resources and associated information, counselling and services. Analysis of the data provision requests from users can be separated into two main categories: (1) availability of improved and reliable metadata, and (2) improved interoperability between databases. These are described below and a more full coverage is given by the project deliverables D8.4 *Report on comparison of various integration software operating in Life Sciences* (https://zenodo.org/record/49968) and D8.6 *Report on human and programmatic access and on documentation development and contents annotation* (https://zenodo.org/record/49970).

## Users’ requests

### Improved and reliable metadata

#### Taxonomy

The use of DNA sequencing has led to numerous taxonomic changes resulting in a large number of species names becoming obsolete and being replaced by newly created ones. However, a large majority of CC users, and specifically those outside the field of taxonomy, do not keep up to date with microbial taxonomy. To best serve the needs of such users, databases should allow for searches using the “old”/obsolete species names in addition to the new ones. Although this practice has been implemented in some Information Systems (e.g., CABRI, http://www.cabri.org/) and web servers (YeastIP, http://genome.jouy.inra.fr/yeastip), it is quite uncommon. In addition, the user community needs integrated tools to allow automatic correction of misspellings in taxonomic names to ensure comprehensive and thorough responses to search queries. There is also a clear need to indicate the various changes in taxonomic nomenclature together with the date of change. Some databases are in the process of implementing such controls, for example, DSMZ (http://www.dsmz.de/), LPSN (http://www.bacterio.net/) and Mycobank (http://www.mycobank.org/). An up-to-date Information System will have to utilize such databases to display the name changes for species already deposited into the CCs.

#### Search range and detail

The need for extensive searches across several databases for multiple characteristics has become the norm and consequently, users of microbial information should be able to execute a wide range of search queries. Improving the quality and diversity of possible searches is a prerequisite for the improvement of services provided by the CC to its user community. One of them concerns the scope of the databases and the metrics on the taxonomic diversity of the CC’s holdings; metadata that is needed to facilitate user searches. The search strings can be simple queries based on the type of strains, the name of the authors who described them or simply by, the isolator of the strain, the depositor, the year of description, the location of isolation, the substrate of isolation, etc. Notably, such simple search queries are rarely implemented in online databases. More complex searches could be used to mine metadata for interesting and novel properties from CC databases that cover temperature growth, ability to assimilate substrates like carbon sources (as found in API^®^ strips), nitrogen sources, ability to ferment various substrates, or specific growth characteristics (thermophily, psychrophily, alcaliphily, aerobic/anaerobic growth, etc.) (Vasilenko et al. 2011). Data fields can also cover passport data, such as substrate and source of isolation including ecological niches, host specificity (plants, animals), etc., as well as more detailed information on specific substrates (oil pollution, vegetative residues, various soils, foodstuff, etc.). More commonly and now a prerequisite is the retrieval of (related) DNA sequences of preserved strains.

Users are also looking for the possibility to search bibliographical references for specific properties of the preserved strains and such mechanisms will be needed in the Information System. Certainly, searching through the possible applications of the microorganism, such as their potential in biotransformation, bioremediation, enzyme and metabolite production, probiotics, etc., are also required. In addition, ecological and metagenomic studies have reinforced the need to search for information on microbial associations. With respect to the ratification of the Nagoya Protocol on Access to Genetic Resources and the Fair and Equitable Sharing of Benefits Arising from their Utilization in October 2014 the information about Access and Benefit Sharing (ABS) agreements as well as Prior Informed Consent (PIC), Mutually Agreed Terms (MAT) and Material Transfer Agreements (MTA) are now essential for any kind of commercial or even not commercial downstream processing. As a corollary, various combinations of the above searches are desirable and, to some extent, needed. For example, taxa and their growth parameters, or recently described taxa with their substrate of isolation. Aside from a few databases, such as the ones at the Centre de Ressources Biologiques de l’Institut Pasteur (CRBIP) at http://catalogue.crbip.pasteur.fr/crbip_catalogue/faces/recherche_catalogue, and the CIRM-Levures at http://genome.jouy.inra.fr/cirm/bdd/, the on-line metadata of other CCs are not organized in a manner that permits searching through a large number of fields simultaneously.

Finally, users are now requiring that the output of searches to be delivered as versatile files or tables that are easy to download for further analysis.

### Improved interoperability between databases

**Fig. 1:**
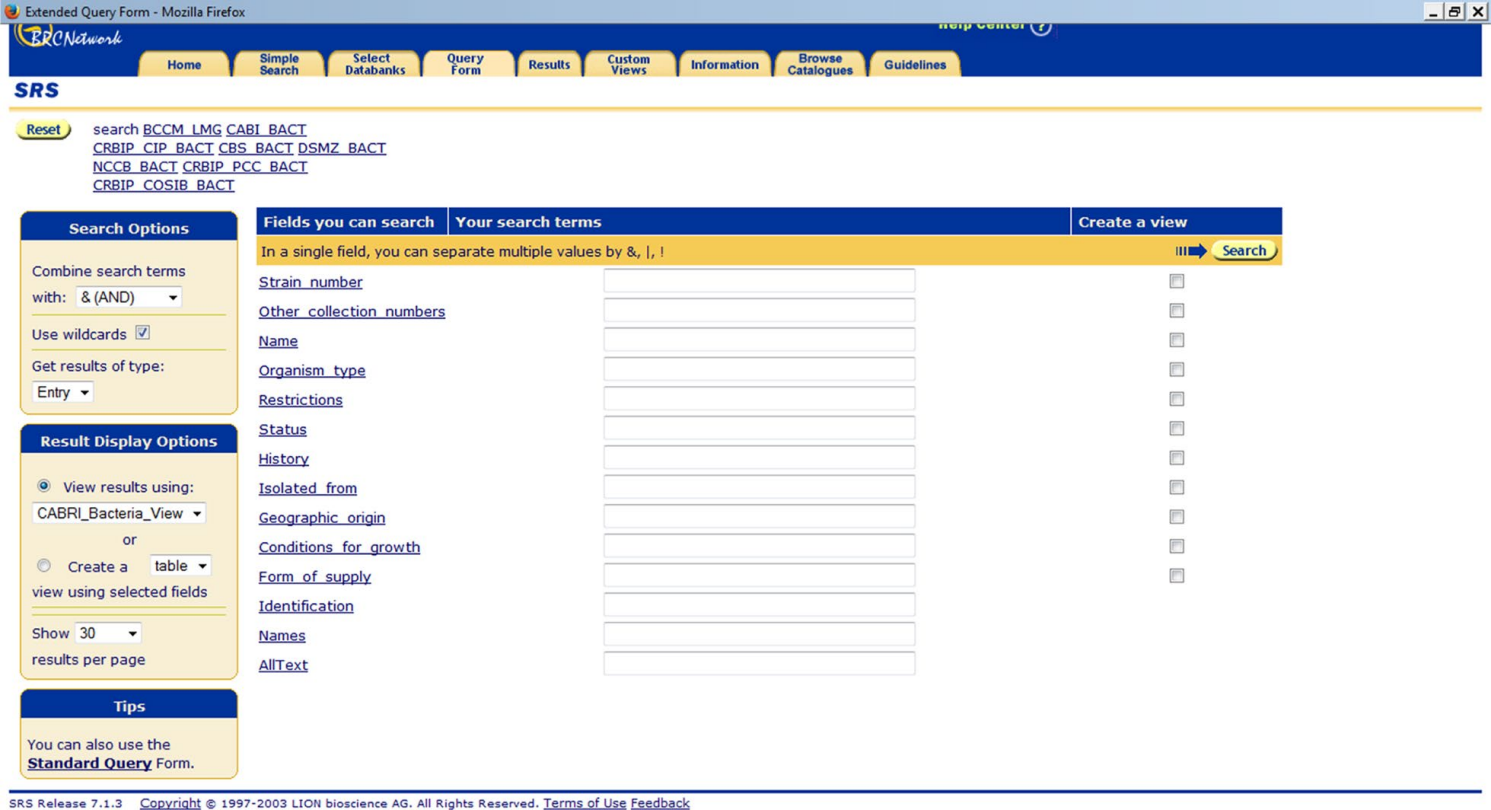
The “Extended Query Form” interface of CABRI. The SRS standard interface allows multiple query conditions to be set combined with boolean operators for each indexed data field. The query form for CABRI bacteria catalogues is shown. A possible response to a query would then return, e.g., all strains of the genus *Phyllobacterium* (field Name), isolated from *Ardisia crispa* (field Isolated_from) in Germany (field Geographic_origin)

There is a large offer of microorganisms and related data from a number of individual CCs, but simultaneously searching their databases presents a challenge. There have been successful endeavours in this regard e.g., CABRI (http://www.cabri.org/) and StrainInfo (http://www.straininfo.net/). The CABRI network services offer integrated access to 25 catalogues including more than 130,000 microbial resources. This was made possible by the definition and adoption of common datasets and a special data format to index the catalogue contents in a coherent manner via the Sequence Retrieval System (SRS, http://www.cabri.org/guidelines/catalogue/CPdata.html) (Etzold et al. 1996). Using the “Extended Query Form” of the standard SRS interface allows searching of all catalogues together and moreover to define multiple query conditions, at best one for each data field (Fig. 1).

**Fig. 2:**
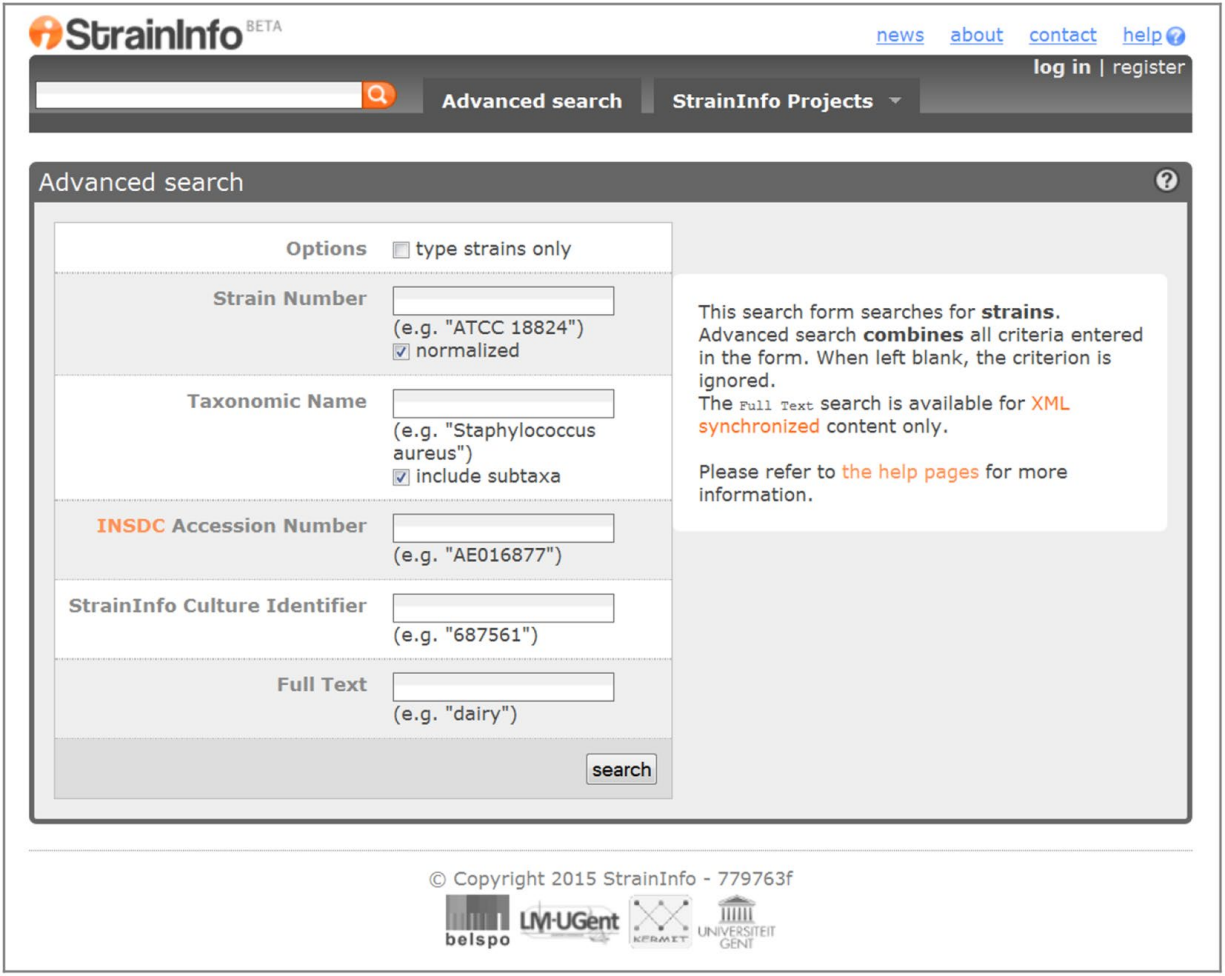
The “Advanced search” interface of StrainInfo. The query form for all the data in StrainInfo is shown. A possible response to a query would then return, e.g., all strains of the genus *Debaryomyces* (field Taxonomic name), isolated from dairy products and their environment (field full text)

The StrainInfo database consists of metadata extracted from CC databases, which was then used to create strain passport data to link the various CC numbers of a given strain to information such as taxonomic name, sequence data, and bibliographic references (Verslyppe et al. 2014). Thus, StrainInfo allows searches for strains belonging to a given species, or searches related to a given strain number, through a large number of databases (64 CCs and BRCs are now referenced in StrainInfo, citing 693,800 culture collection accession numbers of 298,124 strains, as indicated at http://www.straininfo.net/stats) (Dawyndt et al. 2005; Verslyppe et al. 2014). Searches may be complex and simultaneously include various requests (Fig. 2).

User feedback also indicates that the provision of combined lists of microbial cultures, from several source collections, in a consistent format would be appreciated as it would facilitate the selection of the most appropriate cultures for their needs, as demonstrated by the two examples cited above. The search for bibliographic references on cultures is also a top priority for users. They wish to sort data on the basis of names of cultures, authors, journal name, year of publication, etc. The possibility to collate and compare data on the same strain in different collections would therefore be of great use, in particular for the curators of CCs. Such searches of all the comparable fields in the various databases would certainly help to curate data and correct mistakes.

## Curators’ requests

In the previous section, we discussed some of the users’ primary requests and needs. In addition, the curators of CCs have specific needs. A good database must facilitate the curator’s work, whilst at the same time facilitate access to the holdings, and related data, of the CCs to their users. In fact, CC curators would desire harmonization in all areas; harmonization that would include the use of Standard Operating Procedures (SOPs), data structures and software tools. This would pave the way to the construction of an integrated CC knowledge system that would allow searching databases on the basis of properties associated with the preserved microorganism.

Some recommendations for a CC database have already been published (WFCC 2010; Technical_Architecture_Group 2008; GBBRCN 2011). However, current work within the MIRRI project has identified a number of remaining problems and issues that limit, or prevent, the extent of harmonization. Possible solutions for these issues are outlined below.

## Proposed solutions for increased interoperability between the existing databases

The harmonization of data standards has been previously attempted for CCs and a basic level of organization is provided by the Organization for Economic Co-operation and Development (OECD) in their best practices for BRCs through Minimum Data Sets (OECD 2007). Good models for a prototype of a MIRRI request system are also present in existing global Information Systems such as CABRI, Straininfo.net, the World Data Centre for Microorganisms, MycoBank, etc. It must be emphasized that, in parallel to the improvement of databases in European CCs, resources and effort must also be invested in the computerization of information. Additionally, if data is to be interoperable between CC databases, and beyond, common formats for data processing operations and network-access tools suitable for most of the CCs, are essential to facilitate knowledge generation. Similarly, shared data standards should be used for all the common types of data in CC databases. This should be accompanied by uniform principles of operation and informative SOPs.

The first coordinated guidelines for data management were proposed by CABRI, which included rules for producing electronic catalogues in a common format (http://www.cabri.org/guidelines/catalogue/CPdata.html). They were adopted by the OECD best practice guidelines for microbial BRCs (OECD 2007) and then further developed by the Global Biological Resource Centre Network (GBRCN) demonstration project (http://www.gbrcn.org/project/final-report.html). The latter project was in collaboration with the European Consortium of Microbial Resources Centres (EMbaRC), which resulted in project deliverable D. NA1.1.2 “Guidelines for optimal formatting/annotation of data related to the biological materials” and included in the revised EMbaRC BRC standard (http://www.embarc.eu/deliverables.html).

There are at least three ways to implement interoperability as far as a network of databases is concerned. The first relies on the use of common software tools for information processing by partner CCs. For example, instituting a common license to use dedicated software for all partner CCs. This approach is rigid since it does not always take into account the specific needs of individual collections, and/or their different exigencies in terms of IT skills and budget. A possible alternative is to develop new software, preferably collaboratively, under an open source environment. The latter would most likely fit the needs of the collections much better, but it could be expensive both in terms of time and effort, as well as put excessive pressure on IT staff of the network’s members. A third, more flexible, alternative, typically adopted in these situations, would consist of defining a proper data interface that allows the interoperability between CC Information Systems and third-party software. It would include a portal designed for the purpose of interrogating CC catalogues through the interfaces of their Information Systems. Providing support to CCs to set up appropriate interfaces for their Information Systems would thus serve as a cost-effective alternative.

Whichever option is chosen to construct the Information System of MIRRI, one has to recognize that CC staff will need to be trained in informatics and data processing. Such training will also require improved communication between microbiologists and IT staff. In particular, there will need to be clear and consistent terminology, which would mean that IT personnel would need to be familiarized with microbiological terminology and of microbiologists with IT terminology. We recognize that CCs that lack sufficient IT personnel and skills will have to be aided in this venture.

## Meta-analyses of the integrated platform

In the preceding sections, we briefly reported on users’ requests, and on potential features, to be implemented in the future MIRRI Information System. In this section we highlight some of the technological and graphical options for certain features of the user interface.

### Harmonization of field content in database

If one needs to search various databases in a network, the search-field content has to be controlled both semantically and syntactically. During the first World Data Centre for Microorganisms (WDCMs) seminar held at Beijing, China, in May 2011, evidence for differences in the description of strain properties in collection data fields were demonstrated. For example, data on one strain of *Aspergillus brasiliensis* (Varga et al. 2007) were extracted from four catalogues with the following related strain numbers: ATCC 9642, CBS 246.65, DSM 63263 and VKM F-1119. A total of 24 data fields for the given strain from each collection were then paired and compared: only two fields in two collections were syntactically identical, i.e., aside from the same content, they also had the same format. There was in fact no significant differences in the reported strain properties in the four catalogues, aside from the syntax in the terms used to describe them. This example shows that a mere problem of field values could potentially render common searches of various databases difficult and incoherent. Hence, the need for harmonization of the type and description of data in a network of databases is evident.

According to the Taxonomic Databases Working Group (TDWG) roadmap and the experience of the Global Biodiversity Information Facility (GBIF) (WFCC 2010; Technical_Architecture_Group 2008), and BioMedBridges (BioMedBridges WP3: ESFRI BMS Standards Description and Harmonization, http://www.biomedbridges.eu/workpackages/wp3), the crucial components for the interoperability of databases are: “community supported vocabularies”; “ontologies expressing shared semantics of data”; “common exchange protocols”, and “persistent identifiers”.

“Vocabularies” refers to information data standards with detailed specifications of content in data fields (controlled vocabularies), in a specified vocabulary format. On the basis of these standards, identical information for a given concept can be inserted in all involved documents. In our case, in all catalogues of the CC partners. “Community supported” refers to the actual support in adopting and maintaining the tool, i.e., the vocabulary, over time. The starting point for the definition of a community-supported vocabulary may be the creation of a list of popular fields and related content in the catalogues of community members. To determine the most used fields, online catalogues of the WDCM/CCINFO collections were compared. In addition, MIRRI partners provided the fields they included in their catalogues. The subsequent elaboration of a list of those fields commonly used by CCs has paved the way to the establishment of a shared list, and to its adoption by the community. This list may be termed “Recommended Datasets” or “Practical Datasets” (PDS), to avoid confusion with previous CABRI definitions.

“Ontologies” allow for the semantics of both textual and factual information to be encoded and expressed, which is however often implicit and therefore unusable by a software tool. An ontology is a well-defined description of all concepts inherent in a given knowledge domain and of the relationships among them. The most informative ontologies include all instances of concepts, i.e., all values that can be validly associated with a concept. These instances can also be expressed by using vocabularies. In bioinformatics, ontologies have many applications, the most important being data validation and data integration. With respect to data validation, software can be developed to allow the checking of values assigned to information described in the ontology. A simple example is the automatic validation of species names on the basis of a special ontology for microbial names. This could be straightforward, e.g., comparing values listed in a catalogue with the list of valid names in a vocabulary. It could also be further articulated, e.g., when assessing the validity of single components of a scientific name [genus, species, approbation, author(s), year] per se, and in conjunction with the other components of the name.

Regarding data integration, the assignment of a given ontological concept to a piece of information, for example in a database, allows semantically correct connections between heterogeneous databases to be established. One possible example relates to the Gene Ontology (GO; http://geneontology.org/), a widely adopted ontology of gene products. It is possible to “annotate” the description of a strain, i.e., to add GO terms that best fit its properties, to establish a potential connection with all databases that use GO. The shared adoption and use of ontologies is therefore an essential prerequisite for data validation and integration in modern Information Systems. Although some data, such as dates, do not strictly need an ontological description, it is important that all specific information have one.

For strain-associated data, special ontologies including all related concepts and their relationships are required, along with lists of instances (vocabularies) that take into account the variety of CC data for each piece of information. An ontology of fungal names, introduced in April 2013 for use in BioloMICS (https://www.bio-aware.com/), covers many online-catalogue data fields. However, updating strain information to a “new taxonomy”, i.e., to current names, is not straightforward. A study carried out by MIRRI partners on a catalogue list of strains belonging to a species demonstrated that it was rare that a name change applies to all strains of this species. For this reason, the least requirement would be a reference to the publication citing the new taxonomy before a name change could be considered. The changes could eventually be implemented, but only after further work, e.g., such as sequencing being carried out when a species is split on this basis.

In Environmental Ontology, community ontology for the concise, controlled description of environments (EnvO), types of soil were compared with soil classifications and almost all the recognized types were absent from the Metagenome and Microbes Environmental Ontology (MEO) (http://bioportal.bioontology.org/ontologies/MEO?p=classes&conceptid=root). In order to verify if this mal-adoption of existing ontologies in the representation of data in CC catalogues applies to other information, MIRRI partners were asked to provide lists of unique values for each field in their catalogues. These values could then be compared to the content of related domain ontologies. The obvious need for ontologies in database networking suggests that where no ontology is available, an appropriate one should be created. This would need to be a joint effort between microbiologists and IT specialists. To this end, a careful evaluation of existing ontologies in biological, agricultural, and biomedical research is needed. This is especially relevant given that the MIRRI Information System should be made interoperable with many other systems that are not strictly linked to microbiology, but that are nonetheless relevant for microbial resources, such as databases of sequences, proteins, enzymes, and chemical compounds. In this context, the Open Biological and Biomedical Ontologies Foundry (OBO; http://obofoundry.org/), the National Center for Biomedical Ontology (NCBO; http://www.bioontology.org/), and the associated BioPortal (http://bioportal.bioontology.org/), which is self-defined as “the world’s most comprehensive repository of biomedical ontologies”, are all of paramount importance. When searching through the BioPortal, various concepts and instances related to microbiology can be found. For instance, the concept of “strain” is present in 33 distinct ontologies. Three examples of the definitions referring to the microbiological concept of a strain are listed in Table 2.

**Table 2:** Definitions of “strain” in BioPortal ontologies referring to the microbiological concept

Ontology	Code	Definition
Experimental Factor Ontology (EFO)	EFO_0005135	A population of organisms that is genetically different from others of the same species and possessing a set of defined characteristics
Semanticscience Integrated Ontology (SIO)	SIO_010055	A strain is a genetic variant or kind of microorganism
Microbial Culture Collection Vocabulary (MCVV)	MCCV_000002	Strain^a^

^a^MCVV is a very limited ontology, including 16 concepts only

CCs can clearly benefit from current definitions to improve their Information Systems for better interoperability and, moreover, the community of CC researchers can offer important and relevant contributions to other interested parties by providing a proper and extended ontology for microbiological concepts.

### Navigation in the information space of microbiology, bioinformatics, biotechnology, agriculture, medicine

The main goal of the user interface of an Information System is, evidently, to provide the users with: (1) facilitated access to the available information about the strains of interest in CCs, and (2) convenient and efficient tools to browse through, and to extract and/or compare, the associated data. In addition, the system should provide a unique interface for the supply of strains and genetic material held either in one collection, or in a number of different collections. The objective would be to make research easier and more efficient via a unique access point, a “One-stop shop”.

**Fig. 3:**
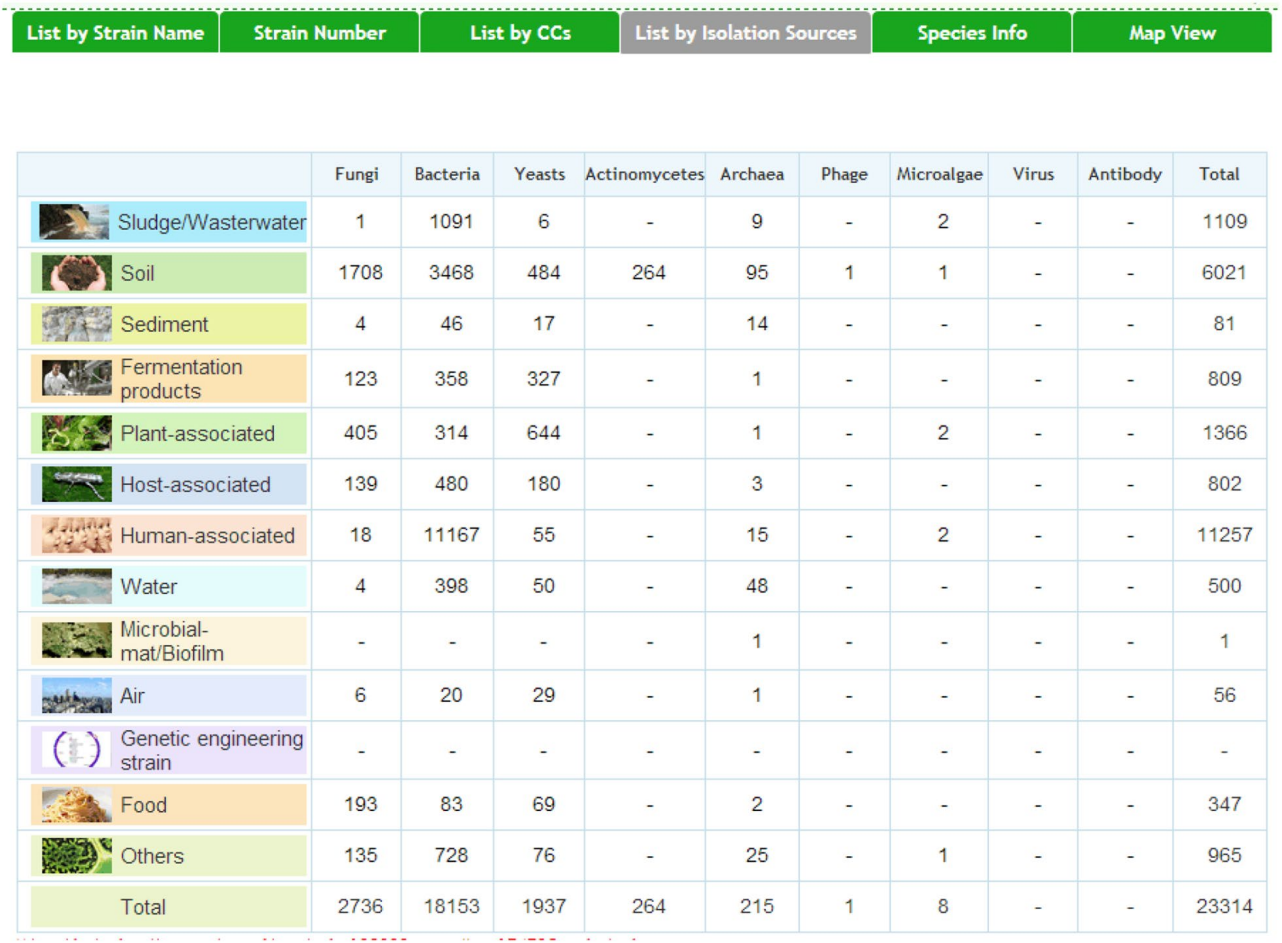
The “Advanced search” interface of WDCM with the request Isolation Source. The result of the request for Isolation Source for the entire content of the GCM (http://gcm.wfcc.info/strains.jsp) 02/02/2015

To date, no structure has been created which fulfils these functions. However, there is one example of a web server, the Global Catalogue of Microorganisms (GCMs) of WDCM, from which a large number of details, such as strain name, strain number, and strains per referenced CC, can be accessed (Fig. 3). Although the GCM and its efficient search portal is an important accomplishment, a number of its features do not cater for the CC users’ needs. The data of each CC needs to be manually transferred to the GCM by the CCs themselves, resulting in a number of out-of-date catalogues. Although advanced, searches are not completely versatile; requests combining more than two fields are not available in the GCM. Nevertheless, the GCM catalogue and its search tools remain the most thorough Information System for microbiological services. This example demonstrates that the key response would be to harmonize the fields in the database network as described above.

**Fig. 4:**
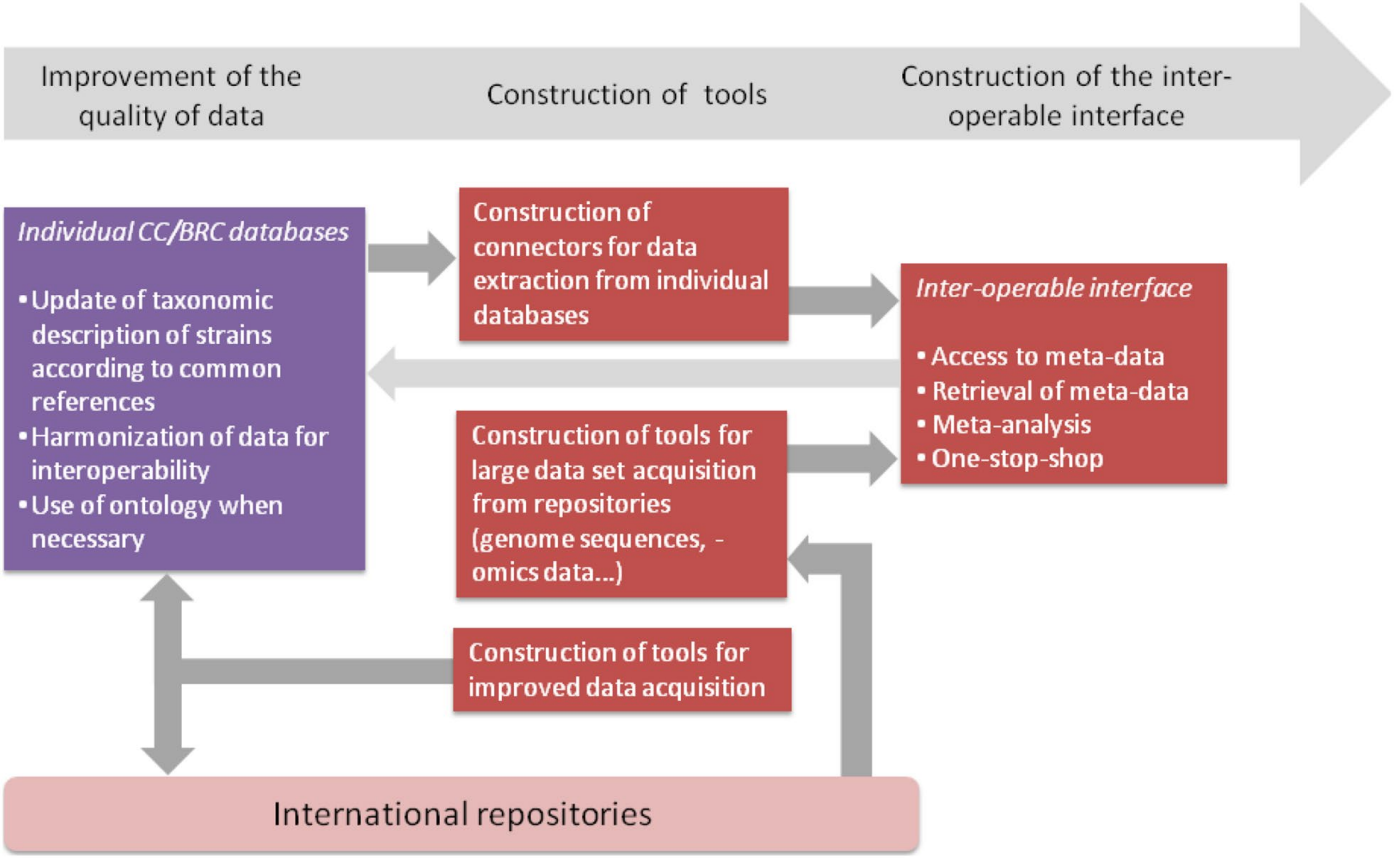
Schematic representation of the main tasks of the construction of the MIRRI-IS. For simplicity’s sake, the hypothesis of an inter-operable interface linking all individual CC databases was chosen (see *Proposed solutions for increased interoperability between the existing databases* section)

An overview of the main tasks involved in the construction of the MIRRI-IS with a temporal perspective is given in Fig. 4.

## Conclusions

A set of basic requirements for a European Information System has been defined after analysing the needs of CC curators and CC users from the information provided by the MIRRI survey, and from discussions at the 2nd WDCM symposium held in Beijing in 2011. As it can be seen from the options and solutions to user needs described here, harmonisation and common tools are required. Data standards and mechanisms for their adoption can be laid down in the MIRRI infrastructure partner charter ensuring conformity and delivery of the envisaged MIRRI Information System. There remain a number of steps to deliver the MIRRI Information System beginning with the evaluation of existing software and tools for its construction. Further work on the scope of the MIRRI Information System will examine interoperability between collections’ data and other relevant data sets outside collections. There is a clear requirement to follow common data management standards, including adopting common ontologies, or vocabularies, if the Information System construction is to be successful. CC staff have developed standards for data management in the EU-FP7 project EMbaRC (European Consortium of Microbial Resource Centres) and in the GBRCN (Global Biological Resource Centre Network). These standards will be assessed in the context of the envisaged MIRRI Information System.

MIRRI must provide the data needed to facilitate the use of microbial diversity in research and development. Further evaluation of the available data is needed to help design mechanisms to link data sets and make the data interoperable. We identified the need to work closely with the ESFRI initiative ELIXIR (European Life Science Infrastructure for Biological Information) and other players such as Straininfo.Net, WDCM and its Global Catalogue of Microorganisms database.

In order to link CC data to other systems it is imperative to respect a certain number of standards so as to allow third parties to use these data and include them in their analyses. In order to do so, software systems used by CCs need to be able to easily, and ideally automatically, export or expose data using a number of structured formats, that are usually XML-based, and are independent of the format of the original database where data are maintained. There are many initiatives trying to establish biological data standards, as well as standards that are used by biologists, such as geographic, climatic or ecological data. Reinventing such standards is certainly not a good idea. What MIRRI would instead do, is “isolate” a number of standards that are relevant to the type of data that CCs are likely to use, and ensure that the software used by CCs are able to produce and utilise such standards. For the first time this report takes into account user needs in the design of a culture collection Information System and lays the foundation for the establishment of the MIRRI Information System.
